# On the Art of Midwifery, as Exercised by Medical Practitioners, in Reply to Dr. Kinglake

**Published:** 1816-04

**Authors:** Samuel Merriman


*For the London Medical and Physical Journal.*

### *On the Art of Midwifery, as exercised by Medical Practitioners, in Reply to Dr. Kinglake; by* Samuel Merriman, M.D. F.L.S.

"Nusquam magis quam in humanâ generatione variat Natura, et in constanti "puerperii actione inconstantiam suam monstrat constantissime."

Th. Bartholin.

THE art of midwifery, as exercised by medical practitioners, has, on various occasions, excited much opposition. In France it was combated by the pious animadversions of Hecquet,* and the declamations of Roussel;† in

---

* M. Hecquet was the original Dr. Sangrado of Le Sage. He was a very humane and pious man. It is reported of him, that he never prescribed in doubtful cases; without first having recourse to prayer. He published "De l'Indecence aux Hommes d'accoucher les Femmes. 1708."—*See Hutchinson's Biographia Medica.*

† M. Roussel was a physician at Montpelier he published "Systeme physique et moral de la Femme. 1775."

England, Dr. Frank Nicholls* attempted to overturn it by sarcastic irony, and the eccentric Philip Thicknesse† by malevolent aspersions; they were assisted in their laudable endeavours by Mrs. Nihell‡ and Mrs. Stephen,§ two midwives, who, though themselves unable to write, could easily procure others to publish in their names against the men-midwives. That these publications, so full of piety, argument, ridicule, and slander, produced but little of the intended effect, is evident, because the practice of midwifery, by men, has been ever since progressively increasing. It may indeed be contended, as these books were very widely dispersed, and consequently must have occasioned a very rigid investigation into the merits of the male and female practitioners, in every point of view, that the question, as to the propriety of employing men, is already virtually set at rest; and that the accoucheurs are only now preferred to the midwives, because they have been proved to possess greater skill, greater judgment, greater mildness, greater patience, and greater decorum. If the men were very deficient in any one of these qualifications, it cannot be believed that they would be employed by the generality of females.

The discussion of this subject has, however, been lately resumed by Dr. Kinglake, who enters the list as the advocate of the midwives, and expresses himself in strong terms against the accoucheurs.

Dr. Kinglake begins his attack by a letter,‖ containing some incontrovertible truths, and not a few erroneous opinions; and this, his first letter, concludes with a rounded period, the gentlemanly expressions of which cannot be too much admired, intimating that midwifery, as at present conducted by men, ought to be abandoned "as a busy, intermeddling, mischievous *craft.*"

This letter may be considered as a splenetic effusion,

* Dr. Nicholls was the author of "The Petition of the unborn Babes to the Censors of the Royal College of Physicians of London; 1751."

† Thicknesse, besides many paragraphs, &c. in the newspapers, published the following virulent pamphlets, "The Danger and Immodesty of employing Men-Midwives, &c. 1772;" "Man-Midwifery analysed; 1775;" "Thoughts on the Times, and the Profligacy of Women; 1779;" "Man-Midwifery dissected, by John Blunt, Surgeon; 1793."

‡ "A Treatise on Midwifery, &c. 1760;" published under the name of Mrs. Nihell; was said to be written by her husband, a surgeon.

§ In her name was published, "The Domestic Midwife, 1795;" which was revised, if not written, by P. Thicknesse.

‖ London Medical and Physical Journal, vol. 34, p. 290.

 which which might have been consigned, without a comment, to its merited oblivion; Mr. Wayte, however, and Mr. Atkinson, did Dr. Kinglake the honour of remarking on it, and their remarks have drawn from the Doctor a long and elaborate replication. This, his second paper,* it is my intention to examine, with the view of ascertaining how far the assertions it contains are correct and candid.

It will not, I conceive, be difficult to shew in the first place, that Dr. Kinglake has undertaken to write upon a subject with which he is very little acquainted; and a passage towards the beginning of his letter may be adduced as a proof. Mr. Wayte having instanced the case of the placenta presenting at the os uteri, as one cause among others requiring the attendance of a scientific practitioner, Dr. Kinglake replies, "With regard to the placental presentation, said to occasionally occur, *it appears to me* that the occasion is as rare as a deviation from the natural course can be; indeed, instead of a deviation from, it may be said to be an inversion of, the order of nature, which is a topsy-turvy course, not reasonably to be calculated on. ......If my information be correct, it is not risking too much to say, that there is *not more than one practitioner in a thousand*, in any age, in any country, that has ever met with an instance of it."

Where Dr. Kinglake sought for *correct* information upon this point, we are left to guess; it is however evident, that his inquiries have not been very extensive. Had he taken the trouble of consulting the collections of cases of Mauriceau, of Portal, of Gifford, of Smellie, or of Rigby, he would have seen sufficient cause to think differently. The last author alone, in his very excellent "Essay on the Uterine Hæmorrhage which precedes the delivery of the full-grown Fœtus," 5th edit. gives the histories of *one hundred and six* women attacked with floodings, of which number *forty-three* were cases of attachment of the placenta over the os uteri. The writer of this letter has, in the last sixteen years, met with *eight cases* of presentation of the placenta among his own patients, and has been called into consultation by other practitioners at least *thirteen times* in this kind of accident. How do these facts agree with the Doctor's surmize, that not more than *one practitioner in a thousand* has ever met with this occurrence?

But Dr. Kinglake goes on to say, "it is not *clear* that a placental presentation, unrelieved and unperforated for the manual delivery of the fœtus, would terminate in death."

It

It may not be clear to those who have paid but little attention to the subject, just as many occurrences in physic are not clear to the illiterate observer, which are, nevertheless, perfectly known to the well educated surgeon or physician. And it is because the case is not clear to the inattentive accoucheur, or midwife, that so many women have lost their lives from this dreadful accident; but the case was clear enough to Ambrose Paré, to Guillemeau, to Mauriceau, to Puzos, to Roederer, to Gifford, to Hunter, to Denman, to Osborn, to Clarke, to Hamilton, and to all the great masters of the obstetric art; and, therefore, they have taken pains to inculcate the important truth, that to preserve the life of the mother, it is necessary to turn the child, and deliver before her strength is too much exhausted. Dr. Denman says decidedly, "this practice is no longer a matter of partial opinion, on the propriety of which we may think ourselves at liberty to debate: it has, for *near two centuries*, met the consent and approbation of *every practitioner of judgment and reputation* in this and many other countries."

Having thus proved that Dr. Kinglake has deceived himself, in supposing that placental presentations are so extremely rare and so easily managed, I shall proceed to shew that he is no less in an error respecting other occurrences in parturition.

So high an opinion has he of the powers of Nature, in effecting the expulsion of the fœtus from the womb, that he seems to consider it scarcely possible for any aid to be required, or to be safely given, during the process of labour; and, in support of this opinion, he says, "It has been ascertained to an extent that sets all questions at rest upon the subject, that medical practitioners, *in full midwifery employ* during upwards of *thirty years*, have never met with an unnatural presentation, have never had an occasion for using an instrument, and have always found the natural efforts equal to all the exigencies of salutary parturition." This is somewhat like the reasoning used by Isaac Massey, the apothecary to Christ's Hospital, and the violent opposer of inoculation, who, to prove that this new practice was unnecessary, argued that the natural small-pox was not of so dangerous a nature as was commonly supposed, for that Sir Hans Sloane, who was the physician to that establishment, had attended *some hundreds* of the children in that disease, and in eight years only lost *one* patient. This one example of singularly good fortune, did not convince the world that the natural small-pox ought not to create alarm; nor will Dr. K.'s singular example prove that purturition is a function always safely performed.

What

What is meant by the term "full midwifery practice," is not very easily understood; it cannot be less than one hundred patients a year, which, in thirty years, amounts to three thousand patients: and the practitioners who have attended three thousand patients without meeting with a single unnatural presentation, and who have always found the natural efforts equal to all the exigencies of the case, must have been fortunate beyond even the most sanguine expectations.

*Midwives* in general are not so fortunate, though Dr. Kinglake thinks that they are better managers of women in labour than the accoucheurs. Dr. Bland, many years the physician-man-midwife to the Westminster General Dispensary, collected from the registers of that charitable institution, a table of accidents and deaths, which happen in consequence of parturition. This table, drawn from 1897 cases of *labours attended by midwives*, was published in the Philosophical Transactions, and demonstrated that

68 of the women had wrong presentations of the children,
12----------------lingering labours requiring instruments,
2----------------convulsions,
9----------------hæmorrhage, of which 3 died,
5----------------the puerperal fever,
2----------------the puerperal mania,
1----------------suppuration of the vagina and bladder,
1----------------a laceration of the perinæum,
5----------------the œdema lacteum; so that, of the whole number, "1 in 18 had preternatural or laborious births, or suffered in consequence of labour," and one case in every forty-four "was attended with particular difficulty or danger."

Really, Dr. Kinglake, by bringing forward these fortunate medical practitioners, of thirty years "full midwifery employ," has done much to prove, that it is safer for women in labour to be attended by men than by midwives.

Perhaps Dr. Kinglake may object to the small number of women, only 1897, from which these averages are drawn: I will, therefore, present him with some other averages drawn from larger numbers; still, however, confining myself to the practice of midwives, because he seems to think that when women attend there is less hazard of a departure from Nature.

Madame Boivin, one of the superintendants of the *Hospice de la Maternité*, at Paris, published, in the year 1812, an account of the various Presentations of the Fœtus which occurred in that Hospital among 12,751 patients; from which it appears, that in 109 cases the child's head was in a wrong direction; direction; in 394 the child presented preternaturally, either with the nates, the feet, the funis, the arm, or in some other way; making, in the whole, one unfavourable presentation in every *twenty-six* labours.

Again, between the 9th of December, 1799, and the 31st of May, 1809, 17,308 women were delivered in the *Maison d'Accouchemens*, at Paris. Here, too, the average of unfavourable presentations was *one in twenty-six;* and in neither of these accounts are the cases of hæmorrhage, of convulsions, &c. at all noticed. It is stated, however, that of the 17,308 women in the *Maison d'Accouchemens*, *two thousand* were afterwards affected with illness, or some serious accident, and that *seven hundred* of them died. The death of *one* woman out of *every twenty-five* delivered, does not very strongly prove the safety of being attended by *midwives.*

Dr. Kinglake alludes to the successful labours of the Asiatic, the African, and the uncivilized American women, who are for the most part left to spontaneous parturition; and "the historians of those people," it is said, "have not cited any of them as instances of suffering for want of the obstetric practice."

That cases of difficult labour are less frequent among these nations than among Europeans, I shall not attempt to deny; that their labours are always unattended with danger, it would be folly to maintain. Many causes concur to render parturition among them more favourable than among us. These women rarely remain unmarried till the parts destined to the functions of generation and parturition become rigid for want of use. Their minds, being less cultivated, are not so easily affected by external causes as is to be found among the European ladies. They eat less of substantial food; and hence, probably, their children at birth weigh less than the children of these climates. They are not so liable to suffer from the rickets in infancy, and hence escape one of the most common causes of difficult labour. Besides which, we learn from the plates of Professor Camper, that the pelvis of many of these women is larger and shallower than the European; and it is well known that their heads are not so bulky. These seem to be the true reasons why the Asiatic, African, and American women suffer less in parturition: it is not, as has been frequently supposed, because their frames are relaxed by the heat of the eastern climate, or their muscles strengthened by the cold of the north. However, as I before remarked, even these circumstances in their favour do not always render their labours safe. Sonnini, a late traveller into the East, admits that difficult labours are now and then met with, by stating the charm charm which is employed to render them easy. Hippocrates states frequent instances of difficulty; and the Bible itself announces several deaths, in consequence of unfavourable parturition.

There is one paragraph in Dr. Kinglake's letter that more especially offends against correctness and candour. He says, "It cannot be heard without shuddering, that *the practice is not rare*, in which, after a lapse of *less than twelve hours* in lingering and inefficient labour-pains, the prompt, the skilful, the instrumental accoucheur denounces the sufficiency of Nature; and, where the presentation is natural, where *no symptoms of imminent danger on the part of the mother have arisen*, he commences his scientific work by boring the fœtal skull, and compressing it within practicable limits for extraction; and when the *ill-judged destructive interference* is over, full credit is asked and given for having saved the mother's life, &c. &c. Here, it is not hastily, not upon the spur of the moment, but calmly and deliberately asserted, that it is "not rare," of course that it is common, for "skilful accoucheurs," in labours "of less than twelve hours" duration, when there are "no symptoms of imminent danger," to perforate the fœtal skull! Is Dr. Kinglake aware of what he has said? Is he aware that he has brought a charge of wilful murder, of murder most foul and most unnatural, against "skilful" medical practitioners? of murder, "not rarely" committed, but systematically adopted, and resorted to again and again, unnecessarily, to, as he adds, "an incalculably baneful extent"?

It is scarcely possible to write upon this disgusting topic with calmness; it requires more than ordinary patience to hear such a calumny advanced against "skilful" medical practitioners: the refutation of the slander is, however, easy; and I hasten to prove, beyond the possibility of dispute, that Dr. Kinglake has, in another instance, shewn himself unacquainted with the subject on which he publishes.

If it were true that the practice alluded to is "not rare," is common, among "skilful" accoucheurs, "to an incalculably baneful extent," how enormous must be the number of still-born children! how much must the column of abortive and still-born, in the Bills of Mortality, be increased beyond the numbers which were known when midwifery was chiefly practised by women!

Well, is this the case? The Bills of Mortality began to be kept accurately in the year 1657, at which time, and for many years after, the practice of midwifery was chiefly exercised by women, and by more skilful midwives than are now usually found, because they were not allowed to practise without a licence, granted after an examination of their abilities, which now is never required. From 1657 to 1681 inclusive, a period of twenty-five years, there were 273,763 christenings and 14,397 abortive and still-born children, so that the children dead-born were to those born alive as 1 to 19. But, during the last twenty-five years, from 1791 to 1815, when the practice of midwifery has been more generally conducted by men, the number of christenings is 492,464 and the still-born children 15,984; which is, to those born alive, as 1 to 30. Notwithstanding, therefore, these atrocious murders, so boldly charged upon the men-midwives, and carried to "such an incalculably baneful extent," it appears, that so far from the practice of midwifery by men having augmented the number of still-born children, it has diminished them more than one-third.

From the same documents, the Bills of Mortality, may be drawn one of the most convincing proofs that can be required of the benefit which the general extension of the obstetric science among men of discrimination and skill has conferred upon the female sex; and this as regards their greater safety in this hour of danger. During the first series of twenty-five years above mentioned, there were, as has been stated, 273,763 christenings, and 14,397 still-born children, making together 288,160 cases of parturition: of these, there died in child-bed 6,686, which is in the proportion of *one in forty-three*. The 492,464 christenings in the second series of twenty-five years, and the 15,984 dead-born children, make a total of 508,448 labours; and, during this period, the number of deaths in child-bed amounts only to 4,684, which is in the proportion of *one in one hundred and eight*. To what cause this diminution of mortality in child-bed is to be attributed, except to the more careful and judicious management of women, in labour and after delivery, adopted by the accoucheurs, I am at a loss to conceive.

But Dr. Kinglake says, though cases may now and then occur requiring the assistance of the accoucheur, or rather of the surgeon, yet the great majority of labours would terminate successfully without his aid. He recommends, therefore, that ordinary cases of parturition should be confided to the midwife, and that the surgeon should be called in only when danger and difficulty present: thus, he thinks, "much less would be heard of preternatural labours, laborious and inefficient efforts for natural parturition, and of resorting to manual and instrumental aid."

I have already shewn, that, under the management of midwives, difficult and dangerous labours are by no means uncommon; and every writer on obstetrics, from the age of Hippocrates to the present, whether practising midwifery as a profession, or only called occasionally to give advice in difficulties and serious accidents, concurs in the same opinion. I apprehend, therefore, that the necessity of the accoucheur is proved from this circumstance alone. But the necessity is likewise proved by the examples, which are numerous in medical records, of the total want of skill and judgment shewn by physicians and surgeons of great general knowledge and acuteness of mind, in cases of irregular and dangerous parturition. Of this a more glaring instance is not to be found than is exhibited in the person of Dr. Kinglake himself. Here is a regularly bred medical practitioner, a man who must have diligently studied anatomy, and all the different branches of the science of medicine, who has been conversant with surgical practice, who has merited and obtained the degree of Doctor of Physic, but is unacquainted with the practice of midwifery: let this gentleman be applied to, in the perilous accident of a placental presentation, he reasons thus—"the uterine contraction that would detach a placenta from the os uteri, would also advance the head of the fœtus sufficiently close to the bleeding source to restrain, by the firm pressure it would occasion, the effusion of blood within safe limits;" and, thus reasoning, he trusts the case to Nature and the midwife: the consequence I will leave him to learn by a perusal of "Mr. Rigby's Essay on Uterine Hemorrhage,"* one of the *latest* publications on the subject; or old Guillemeau's chapter, "*Du Moyen de secourir la Femme en son travail, estant accompagné de Flux de Sang*," one of the earliest.

Unless the practitioner be very conversant with the whole process of natural labour, and of labours unattended with danger, it is impossible that he can distinguish, in difficult cases, when and how to give the requisite artificial assistance: hence the necessity of having a class of men properly educated for the practice of midwifery, and in the constant exercise of that branch of the medical profession. To expect that physicians or surgeons, not habitually practising midwifery, should be able to determine in difficult emergencies, what mode of practice ought to be adopted; or, having determined that artificial aid was required, that they should know how, either by the hand or by instruments, to give the necessary assistance, is not less absurd than it would be to call into consultation, in an obscure and complicated malady, the physician who had not yet attended the more common diseases; or to require the surgeon, not habituated to perform minor operations, to undertake those that are technically termed Capital.

* I particularly recommend him to read with attention, Cases x, xiv, xv, xx, lxxxi, xcviii, and ci.

I might now expatiate upon some expressions in Dr. Kinglake's letter, which do not seem to be mere slips of the pen—but I hold my hand; he who can boast of using "harsh epithets," because he thinks "the cause in which they are used is that of philosophical truth, which disdains blandishments, and expresses itself in unequivocal firmness," would hardly be convinced by any arguments I could employ, that the cause of "philosophical truth" is more likely to be promoted by moderate language and gentlemanly expletives.

*Half-Moon-street; March* 14, 1816.

---

*For the London Medical and Physical Journal.*

